# Family time, parental behaviour model and the initiation of smoking and alcohol use by ten-year-old children: an epidemiological study in Kaunas, Lithuania

**DOI:** 10.1186/1471-2458-6-287

**Published:** 2006-11-23

**Authors:** Asta Garmienė, Nida Žemaitienė, Apolinaras Zaborskis

**Affiliations:** 1 Department of Social Paediatrics, Institute of Biomedical Research, Kaunas University of Medicine, Kaunas, Lithuania

## Abstract

**Background:**

Family is considered to be the first and the most important child development and socialization bond. Nevertheless, parental behaviour model importance for the children, as well as family time for shared activity amount influence upon the child's health-related behaviour habit development has not been yet thoroughly examined. The aim of this paper is to indicate the advanced health-hazardous behaviour modelling possibilities in the families, as well as time spent for joint family activities, and to examine the importance of time spent for joint family activities for the smoking and alcohol use habit initiation among children.

**Methods:**

This research was carried out in Kaunas, Lithuania, during the school year 2004–2005. The research population consisted of 369 fifth-grade schoolchildren (211 (57.2%) boys and 158 (42.8%) girls) and 565 parents: 323 (57.2%) mothers and 242 (48.2%) fathers. The response rate was 80.7% for children; 96.1% and 90.6% for mothers and fathers correspondingly.

**Results:**

Eating a meal together was the most frequent joint family activity, whereas visiting friends or relatives together, going for a walk, or playing sports were the most infrequent joint family activities. More than two thirds (81.5%) of parents (248 (77.0%) mothers and 207 (85.9%) fathers (p < 0.05)) reported frequenting alcohol furnished parties at least once a month. About half of the surveyed fathers (50.6%) together with one fifth of the mothers (19.9%) (p < 0.001) were smokers. More frequently than girls, boys reported having tried smoking (6.6% and 23.0% respectively; p < 0.001) as well as alcohol (31.16% and 40.1% respectively; p < 0.05). Child alcohol use was associated both with paternal alcohol use, and with the time, spent in joint family activities. For instance, boys were more prone to try alcohol, if their fathers frequented alcohol furnished parties, whereas girls were more prone to try alcohol, if family members spent less time together.

**Conclusion:**

Joint family activity time deficit together with frequent parental examples of smoking and alcohol use underlie the development of alcohol and smoking addictions in children to some extent. The above-mentioned issues are suggested to be widely addressed in the comprehensive family health education programs.

## Background

Family is considered to be the first and the most important child development and socialization bond. Harmonious child-parent interaction, open communication, and parental support are the factors that underlie a successful child's mental and physical health development [1]. Family time devoted for shared activities preconditions a successful communication within the family [2,3].

Nowadays families, as the society itself, are affected by rapid changes. An ever-increasing rush brought on by the information age influences the earlier-settled interaction of the family members, and alters the communication patterns between parents and children. A margin between work and leisure is notably disappearing, and an ever-increasing number of people are never completely free from work [4]. The above-mentioned fact seems to have a direct influence upon the quality as well as the quantity of family time. Some research data demonstrate that family members that are more frequently engage in family activities, increase their marital satisfaction, and enhance the stability of their family [5,6]. When families get involved in joint activities, their children do better at school and grow up to become more successful adults [7]. Several studies have demonstrated a substantial and consistent relationship between family time and habits of smoking and drinking, drug abuse and sexual intercourse experience, and delinquent behaviour in children [8-10].

Parents are the preferential and the most important behavioural development models for children. The quality of this primary social bond underlies the development of the child's future well-being, problem behaviour, and emotional disturbances. [11,12]. Nevertheless, parental behaviour model importance for the children, as well as family time for shared activity amount influence upon the child's health-related behaviour habit development has not been yet thoroughly examined. The aim of this paper is to indicate the advanced health-hazardous behaviour modelling possibilities in the families, as well as time spent for joint family activities, and to examine the importance of time spent for joint family activities for the smoking and alcohol use habit initiation among children.

## Methods

This cross-sectional research constitutes the fourth stage of a long-term epidemiological study, launched in 1999. The study aims at young people lifestyle and health related behaviour follow-up investigation of six-year-old children until the end of their adolescence. The study intervals coincide with the main child social life periods, that is, the kindergarten, primary, and secondary school. The original sample of 577 six-year-olds was randomly selected at 12 kindergartens in Kaunas, Lithuania. Every separate child social life stage implies focusing on different child health and social well-being aspects. The current research findings were collected during the years of 2004–2005, when the investigative children were entering the adolescence and starting to attend secondary schools.

### Participants and study procedures

The research was coordinated by the Laboratory for Social Paediatrics at the Institute for Biomedical Research, Kaunas University of Medicine. The research ethical clearance, as well as the support together with all relevant information was obtained from Kaunas Regional Bioethical Committee of Biomedical Research, Department of Education at Kaunas municipality and the administrations of schools. Parent written consent to take part in the investigation was also obtained.

The research was performed at 41 schools in Kaunas, Lithuania. The investigation material consisted of a structured questionnaires, filled in by 369 fifth-grade schoolchildren (211 (57.2%) boys and 158 (42.8%) girls; response rate 80.7%), aged 10, as well as 565 parents (323 (57.2%) mothers (response rate 96.1%) and 242 (48.2%) fathers (response rate 90.6%)). During the research period, 267 fathers and 336 mothers lived together with the investigative sample of the 369 children. The response rates for fathers and mothers were individually calculated according to the number of the distribution of both sexes parents in the families.

### Measures

With an aim to obtain authentic research data, the survey instruments were kept confidential. Repetition of the equally structured questions in the questionnaires for both parents and children allowed of the parent and child response relation. In order to assess the substance use prevalence among the family members, schoolchildren were addressed with 5, and parents (fathers and mothers) were addressed with 3 questions regarding smoking and alcohol consumption **[See **Additional file 1**, Questions]**. The schoolchildren answers allowed us to determine the tobacco smoking and alcohol consumption onset age, as well as smoking frequency. Fathers and mothers were inquired about the smoking frequency, as well as about the participation in situations with alcohol use. Daily smoking was an indicator for selecting the regular smoker group. The familial alcohol consumption frequency was assessed on the grounds of parental responses to a question: "How often do you participate in alcohol-furnished parties, where you do consume alcohol, even though the least amount of it?" Six alternative response options were suggested: 1) 'almost every day'; 2) 'about 2–3 times a week'; 3) 'about once a week'; 4) 'about once a month'; 5) 'about once a year'; 6) 'never'. The respondents were subdivided into three groups according to the alcohol consumption frequency:

Group1 included the ones, stating frequenting alcohol furnished parties at least once a week and more often. This Group1 was comprised of the respondents, who chose the responses from 'almost every day', to 'about 2–3 times a week', and 'about once a week'.

Group2 included the ones, stating frequenting alcohol furnished parties at least once a month. This Group2 was formed of the respondents, who chose the response 'about once a month'.

Group3 included the ones, who stated seldom participating in alcohol furnished parties. This Group3 was comprised of the respondents, who chose the responses from 'about once a year' to 'never'.

The current research was carried out employing the same family time evaluation methodology as it was employed in the WHO Cross-National Study of Health Behaviour in School-Aged Children (HBSC) [13]. The family time indicator was calculated according to the frequency of collectively spent family time for joint activities. Children were asked to answer the following question: "How often do you and your family together do each of these things?" The suggested responses were as follows: a) 'watch TV or a video'; b) 'play indoor games'; c) 'have a meal'; d) 'go for a walk'; e) 'go places'; f) 'visit friends or relatives'; g) 'play sports'; h) 'sit and talk about things'. Each response was assigned a value from 1 (least family time), to 5 (most family time) scores according to the quantity of the family time allocated: ^'^every day' = 5, 'most days' = 4, 'about once a week' = 3, 'less often' = 2, 'never' = 1.

### Statistical analysis

Statistical Package for Social Sciences (SPSS) for Windows (version 13.0) software was used to conduct data analysis. The statistical relationship between qualitative variables was assessed by Chi-square test, with significance level 0.05 and odds ratio with 95% confidence interval (CI). Family time indication mean values and standard deviations were calculated. The higher mean values, the higher probability for the certain activity to be performed together with the family.

In order to assess the family time in general, and to be able to present the current research, all the above-mentioned eight joint family activity indications were combined into one derivative variable, labelled "Family Time Index" (FTI). The factor analysis was employed to calculate the index [14], and provided a possibility to estimate the extent of each item in the linear combination of the items, as well as proved to be a more rigorous scientific method than a simple sum of the family time indication scores. The values for FTI appeared to be distributed within the range of -3.17 and 2.72. Based on the FTI values, the family time was grouped into two groups. Positive FTI values were typical of the families, where the members reported more commonly-spent time (indicated as 'good family time'); and, vice versa, negative FTI values demonstrated that families tended to spend less time together (indicated as 'poor family time'). The research finding revealed 41.6% of families with a 'good family time' indication; while 58.4% of families were indicated with a 'bad family time'.

A logistic regression analysis was computed with an aim to ascertain the risk of child smoking and alcohol use initiation, in relation to a set of predictors, including family time.

## Results

### Smoking and alcohol use

The parental smoking habit assessment revealed 11.5% of families with both parents being smokers, 43.8% of families with one parent being smoker, and 44.7% of families with no parents being smokers. Father came out to be a more frequent smoker than mother was (50.6% and 19.9% respectively; p < 0.001). In a group of smokers, 89.0% of fathers, and 63.2% of mothers smoked regularly.

The familial alcohol consumption frequency was assessed on the grounds of parental responses to a question: "How often do you participate in alcohol-furnished parties, where you do consume alcohol, even though the least amount of it?" 77.0% of mothers, and 85.9% of fathers (p = 0.008) reported participating in these kinds of parties at least once a month.

The research revealed 55 (15.9%) smoking attempting children (more frequently boys than girls; 23.0% and 6.6% respectively; p < 0.001). The mean of the age of the onset of smoking equalled to 8.59 ± 0.29 years of age. The findings revealed 1.2% of smoking schoolchildren, all of them being boys.

A group of 123 (36.2%) children reported having tried alcohol. The percentage of alcohol consumption attempts admitting respondents was higher among boys than among girls (40.1% and 31.1% respectively; p = 0.086). The mean of the age of the onset of alcohol consumption attempts among children equalled to 8.80 ± 0.17 years of age. Twenty (5.9%) respondents (7.0% of boys, 4.7% of girls; p = 0.392) reported having experienced being drunk.

The statistical analysis indicated a relationship between the mother's and the son's smoking habits. 19.8% of sons of the non-smoking mothers, and 38.2% of sons of the smoking mothers had tried smoking themselves (Table 1). The odds ratio (OR) calculation revealed the smoking mothers' sons being 2.5 times more likely to start smoking, if compared with non-smoking mothers' sons. However, father's smoking seemed to have no effect on children smoking attempts.

The periodicity of parental alcohol furnished party frequenting was related to the onset of alcohol use among boys (Table 2).

The first alcohol use experience frequency was increasingly interrelating with the reported parental alcohol use frequency at the alcohol furnished parties. 8.3% of boys from the families where both parents were reluctant to frequent alcohol furnished parties, 31.6% of boys from the families where at least one of the parents tended to frequent alcohol furnished parties (for comparison with the first group of the parents, OR = 5.08; p = 0.160), and 44.6% of boys from the families where both parents were prone to frequent alcohol furnished parties, reported having attempted alcohol use (OR = 8.84; p = 0.041). Nevertheless, the above-mentioned pattern could not be established in the group of girls.

### Family time

The findings revealed that eating a meal together was the most frequent joint family activity, whereas visiting friends or relatives together, going for a walk, or playing sports were the most infrequent joint family activities (Table 3).

Eating a meal with parents (p = 0.014) was a more frequent joint family activity among girls, while playing sports with parents was a more frequent joint activity among boys (p < 0.001). Nevertheless, no gender differences were observed in other shared activities.

In order to assess the correlation between the joint family activity items, the correlation matrix was verified. The highest correlation values were revealed among such indications, as 'going places', 'going for a walk', and 'playing at home'. The indication of 'watching a TV or a video' was least associated with other shared activity indications.

### Family time, smoking and alcohol use

**Figure 1:**
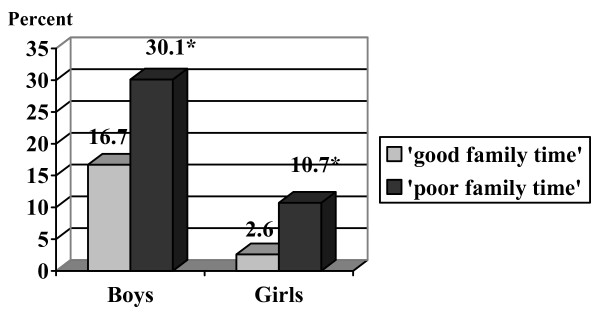
**Percentage of children with smoking attempts according to family time amount**. *p < 0.05 in comparison with 'good family time'.

Family time indication was revealed to be highly associated with both smoking and alcohol consumption by either children or parents; however, the nature of the relationship remains ambiguous. The highest 'poor family time' indication prevalence was observed in the group of children with smoking attempts, consequently suggesting an assumption that smoking-attempting boys and girls come out to be less involved in shared familial activities. Correspondingly, less of the collectively spent family time indicating children were more prone to try smoking (Fig. 1).

**Figure 2:**
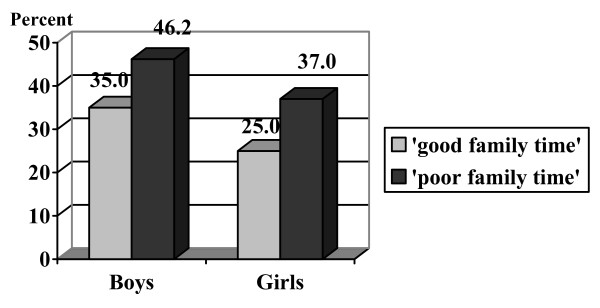
**Percentage of children with alcohol use attempts according to the family time amount**. * p < 0.05 in comparison with 'good family time'.

In comparison with children, who spent more time with their parents (indicated as 'good family time'), the boys, who spent less time with their parents (indicated as 'poor family time'), were 2.15 times more inclined to start smoking (95% CI 1.09 – 4.27). The girls, who spent less time with their parents (indicated as 'poor family time'), in comparison with children, who spent more time with their parents (indicated as 'good family time') were 4.48 times more inclined to start smoking (95% CI 0.92 – 21.82). Analogous, although weaker, association was detected among children with attempts to try alcohol (Fig. 2).

There has been a strong relationship determined between the collectively spent family time and parental smoking and alcohol use habits. For example, mothers from the families, were 'good family time' was indicated, reported generally smoking less frequently, if compared to mothers from the families, where 'poor family time' was indicated (15.0% and 23.7% respectively; p = 0.049).

The findings revealed the development of child smoking and alcohol use habit formation being closely related to parental smoking and alcohol use example frequency, together with the fact of less collectively spent family time. Therefore, the need for determining the highest inside family surveyed impact-making factors upon the children smoking and alcohol use initiation was inevitable. A logistic regression analysis allowed measuring the relationship between the following factors and the child smoking and alcohol use habit formation, in case of the simultaneous impact of the above-mentioned factors (Table 4).

The analysis of the findings revealed the onset of smoking among boys being significantly dependent upon the collectively spent family time amount. In comparison with the boys from the families with an indicated 'good family time', the boys from families with an indicated 'poor family time' were 3.03 times more inclined to try smoking. According to the analysed data, parental smoking, as such, makes an insignificant impact. (Based on the small number of those, who have tried smoking, the analogous analysis was not applied to the group of girls.)

Father alcohol furnished party frequenting made an essential impact on boy alcohol initiation. This indicator was statistically significant for all alcohol consumption periodicities, starting from 'once a week', to 'more often', or 'only once a month'. Collectively spent family time amount made no impact on boy alcohol initiation; however, it influenced girl alcohol initiation. After applying the one-level and multilevel analysis, the daughters of the alcohol furnished party once a month frequenting mothers were proved to be less prone to try alcohol (Table 4). This indicator odds ratio was less than 1 (OR = 0.19; p = 0.031).

## Discussion

The research highlights a familiar notion of child's psychological resistance and vulnerability onset being traced back to family circumstances. As the first child's social environment, family provides the child with a background for attitudes and values, presents with first and the most important life models as well as communication skills. An adolescent child triggers new family development stages. Adolescents shift their social concern onto their peers, and, consequently, family has to recognize the changes as well as the altered relationship between parents and children [15,16]. In search of their identity, adolescents experiment with various behavioural models, including the health-hazardous ones. Young people substance use is becoming a worldwide problem; an use onset occurring at ever-younger ages. Current surveys indicate a steady increase in smoking and alcohol use trends among Lithuanian adolescents during the last decade [13,17]. According to the data of the WHO Cross-National Study on Health Behaviour in School-aged Children (HBSC), the prevalence of regular alcohol consumption among schoolchildren has increased from 9.4% to 13.6% among boys, and from 4.2% to 6.5% among girls during the study period (1994 – 2002) [18]. Smoking prevalence among teenagers has increased 5 times during the last decade; now smoking teenagers outnumber adult women [19,20].

Despite the fact of family contribution in child development being investigated in various aspects, the transitional adolescent period implication, as well as the parent-children relationship type, both influencing the health-hazardous child behaviour development, has been poorly analyzed. The current survey provides an opportunity of the first adolescent children risk behaviour manifestation classification as well as of the first harmful habit development assessment on the grounds of the two important behavioural development aspects, that is, the analogous parental behaviour model, and the amount of family time for shared activities.

The current research data are highly representative for the Lithuanian population, and serve as main socio-economic family status indicators; however, the study sample was drawn from Kaunas habitants only. The sample size and response rate were adequate for estimated relationship statistical significance. The study instrument for assessing the family time for shared activities maintained appropriate internal and external validity, test-proven by the authors of the instrument [13]. Moreover, the research presents one-stage findings out of a long-term epidemiological young people lifestyle and health related behaviour follow-up investigation of six-year-old children until the end of their adolescence. Repetitive contacts with the participants during the clearance of the investigation aims as well as the long-term investigation itself encouraged the children and the parents for an active and open participation in the investigation. The given analysis does not include a comparison in a long-term perspective, therefore should be considered as a cross-sectional study of a randomly selected population. The further analysis of the first smoking and alcohol use manifestation in adolescence might include variables that had been measured in the previous stages of this longitudinal study.

The parental behaviour model importance for the children was substantiated and proven on the grounds of different research and experiments [21-25]. Children evidently do not only soon become aware of the parent habits (smoking, drinking alcohol); they also notice even the slightest details of their parent behaviour. The available data demonstrate that smoking parents' children attempt smoking more frequently, if compared to the non-smokers' children [26-28].

According to the current research data, families with non-smoking parents, and families where at least one of the parents was a smoker could comprise two similar-sized groups. Despite the fact that mothers smoked twice less frequently (if compared to fathers), mother smoking was statistically significantly related to their son smoking attempts. Maternal smoking (especially during pregnancy) may not only serve as a behavioural model; it may physically precondition the child demand in nicotine stimulation [29]. Similar observations concerning the enhanced maternal, rather than paternal smoking influences upon the child smoking habits are also featured in other publications [30-32]. However, the unambiguous opinion, whether maternal or paternal smoking has a more significant modulating effect upon the child behaviour, is still unavailable, due to the fact that data solely confirm a major paternal smoking influence [33]. Presumptively, the effect intensity is preconditioned by the parent gender and methodological survey differences, as well as by the cultural environment and traditional male and female family roles.

Certain behavioural model exposition frequency may become a significant health-related behaviour acquisition process factor. From the authors' point of view, the latter factor could be viewed as one among the possible explanations of the detected relationships between the child and parent alcohol use examples. The incidence rate of boys with alcohol use attempts increased correspondingly with the increasing frequency of parental participation in alcohol-furnished parties.

The fact that the smoking and alcohol use onset was higher among boys than among girls might be viewed as influenced by socialization regarding gender differences, and the determined tendency towards external behavioural problems in boys. [34].

The data regarding psychological family climate and child-parent communication are abundant [1,35]. Unfortunately, there is little scientific material available on one of the principle items of communication, that is, the joint activities of parents and children. The last decade has brought about substantial changes in the family behaviour pattern causing the shift in family models and the increase in the number of divorces, which, in turn, have a negative influence upon the communication between children and parents [1,35]. Single-parent family household incurs high demands on a single biological parent, forcing him/her to work more in order to support the family, thus leaving less time for the children [36]. The fact of a high time deficit for the children was confirmed by different international investigations. According to the recent US survey, two out of three parents stated that in case they had more free time, they would spend it with their children [37]. The results of the current survey reflected the familiar tendencies, namely, two-thirds of the schoolchildren have daily meals with their parents, and about one third of them have a daily possibility to talk with their parents about various things. Children and adolescents, who spend less time with their parents, are more susceptible to the development of risk behaviour [38]. The current research data confirm the above-mentioned fact that adolescents, who share less time with their parents in joint activities, are more prone to try smoking as well as alcohol.

The results of the current survey revealed the parental behaviour, as well as child-parent communication influence upon the child's behavioural development. The risk behaviour prevention implementation among schoolchildren require the highlighting of the importance of family time devoted to shared activities for the child's health development. The education of parents, the promotion of their motivation in favour of healthy lifestyle, as well as encouragement to spend more time with the children can reduce the manifestations of risk behaviour among the adolescents.

## Conclusion

Joint family activity time deficit together with frequent parental examples of smoking and alcohol use underlie the development of alcohol and smoking addictions in children to some extent. The above-mentioned issues are suggested to be widely addressed in the comprehensive family health education programs.

## Competing interests

The author(s) declare that they have no competing interests.

## Authors' contributions

AG carried out the investigation, performed the statistical data analysis, participated in the data interpretation, and drafted the manuscript. NZ substantially contributed to the investigation project and design development, participated in the data interpretation and manuscript preparation. AZ coordinated the investigation, helped to perform the statistical analysis as well as to prepare the draft of the manuscript. All authors read and approved the final manuscript.

## Pre-publication history

The pre-publication history for this paper can be accessed here:



## Supplementary Material

Additional file 1 We presented the questions used in the survey for the assessment of the advanced health-hazardous behaviour modelling possibilities in the families, as well as time spent for joint family activities, and for the examination of the importance of time spent for joint family activities for the smoking and alcohol use habit initiation among children. Click here for file

## Figures and Tables

**Table 1:** Mother smoking relationship to son smoking attempts

Variables and Their Categories	Sons (never tried smoking)	Sons (tried smoking)	Total	Statistical Accounts	
				
				OR	95 % CI
Non-smoking Mothers	105 80.2%	26 19.8%	131 100%	1	-
Smoking Mothers	21 61.8%	13 38.2%	34 100%	2.50*	1,11 – 5,64

Total	126 76.4%	39 23.6%	165 100%	χ^2 ^= 5.06; df = 1; p = 0.025	

* OR is significantly (p < 0.05) greater than 1

**Table 2:** Mother and father alcohol furnished party frequenting relationship to children alcohol use attempts

Variables and Their Categories	Children (never tried alcohol)	Children (tried alcohol)	Total	Statistical Account	
				
				OR	95% CI
*Boys:*					
Mothers seldom participating in parties	33 75.0%	11 25.0%	44 100%	1	-
Mothers participating in parties once a month	52 57.8%	38 42.2%	90 100%	2.19	0.99 – 4.88
Mothers participating in parties at least once a week	14 48.3%	15 51.7%	29 100%	3.21*	1.19 – 8.72
Total	99 60.7%	64 39.3%	163 100%	χ^2 ^= 5.97; df = 2; p = 0.049	
Fathers seldom participating in parties	14 93.3%	1 6.7%	15 100%	1	-
Fathers participating in parties once a month	32 59.3%	22 40.7%	54 100%	9.62*	1.18 – 78.5
Fathers participating in parties at least once a week	29 53.7%	25 46.3%	54 100%	12.06*	1.48 – 98.3
Total	75 61.0%	48 39.0%	123 100%	χ^2 ^= 7.87; df = 2; p = 0.020	

*Girls:*					
Mothers seldom participating in parties	17 60.7%	11 39.3%	28 100%	1	-
Mothers participating in parties once a month	69 75.8%	22 24.2%	91 100%	0.49	0.20 – 1.21
Mothers participating in parties at least once a week	7 58.3%	5 41.7%	12 100%	1.10	0.28 – 4.37
Total	93 71.0%	38 29.0%	131 100%	χ^2 ^= 3.40; df = 2; p = 0.183	
Fathers seldom participating in parties	13 76.5%	4 23.5%	17 100%	1	-
Fathers participating in parties once a month	41 68.3%	19 31.3%	60 100%	1.51	0.43 – 5.23
Fathers participating in parties at least once a week	14 70.0%	6 30.0%	20 100%	1.39	0.32 – 6.08
Total	68 70.1%	29 29.9%	97 100%	χ^2 ^= 0.42; df = 2; p = 0.811	

* OR is significantly (p < 0.05) greater than 1

**Table 3:** Response^† ^means and standard deviations (questioned on family time for shared activities)

Shared Activity	Percentage of the answers "every day"	Mean^†^	Standard Deviation
Watching TV or a video	29.9	3.67	1.16
Playing indoor games	13.2	3.03	1.18
Eating a meal	74.5	4.57	0.88
Going for a walk	9.2	3.00	1.07
Going places	11.9	3.34	0.99
Visiting friends or relatives	4.6	2.86	0.86
Playing sports	9.4	2.62	1.24
Sitting and talking about things	37.5	3.81	1.22

^†^Response codes: every day = 5, most days = 4, about once a week = 3, less often = 2, never = 1

**Table 4:** Family time and parental addiction importance for the child addiction development: multilevel logistic regression analysis results

Dependent Variable	Independent Variables	OR	95% CI	p
*Boys:*				
Tried smoking	'Poor family time'^†^	3.03*	1.15 – 7.96	0.025
	Smoking mothers^‡^	1.38	0.44 – 4.29	0.583
	Smoking fathers^††^	1.28	0.50 – 3.28	0.608

Tried alcohol	'Poor family time'^†^	1.64	0.76 – 3.53	0.207
	Mothers participating in parties once a month ^‡‡^	1.08	0.36 – 3.22	0.893
	Mothers participating in parties at least once a week ^‡‡^	1.99	0.51 – 7.79	0.323
	Fathers participating in parties once a month ^‡‡^	9.23*	1.02 – 83.7	0.048
	Fathers participating in parties at least once a week^‡‡^	10.43*	1.11 – 98.1	0.040

*Girls:*				
Tried alcohol	'Poor family time'^†^	3.35*	1.22 – 9.21	0.019
	Mothers participating in parties once a month^‡‡^	0.19*	0.04 – 0.86	0.031
	Mothers participating in parties at least once a week ^‡‡^	0.36	0.05 – 2.54	0.307
	Fathers participating in parties once a month ^‡‡^	4.75	0.75 – 30.1	0.098
	Fathers participating in parties at least once a week ^‡‡^	4.34	0.57 – 32.9	0.155

^† ^in comparison with 'good family time'

^‡ ^in comparison with non-smoking mothers

^†† ^in comparison with non-smoking fathers

^‡‡ ^in comparison with mothers/fathers seldom participating in alcohol-furnished parties

* OR is significantly (p < 0.05) less or greater than 1
