# Supplementary material for: Family time, parental behaviour model and the initiation of smoking and alcohol use by ten-year-old children: an epidemiological study in Kaunas, Lithuania
Source: BMC Public Health. 2006 Nov 23;6:287. doi: 10.1186/1471-2458-6-287 (PMC1665457; doi:10.1186/1471-2458-6-287)
Supplement: Additional file 1 — We presented the questions used in the survey for the assessment of the advanced health-hazardous behaviour modelling possibilities in the families, as well as time spent for joint family activities, and for the examination of the importance of time spent for joint family activities for the smoking and alcohol use habit initiation among children. [file 1471-2458-6-287-S1.doc]

**Schoolchildren’s questionnaire**

Have you ever smoked tobacco? (At least one cigarette, cigar or pipe)

1. Yes
2. 2. No

How often do you smoke tobacco at present?

1. Every day
2. At least once a week, but not every day
3. Less then once a week
4. I do not smoke

Please indicate the age in what being you have tried smoking (more then one puff)?

I was.................year old

Please indicate the age in what being you have tasted alcohol (more then one drink)?

1. I was.................year old
2. I have never tasted alcohol

Have you ever had so much alcohol that you were really drunk?

1. No, never
2. Yes, once
3. Yes, 2-3 times
4. Yes, 4-10 times
5. Yes, more than 10 times

How often do you and your family usually do each of these things all together? Please tick one box for each line.

|  | *Every day* | *Most days* | *About once a week* | *Less often* | *Never* |
| --- | --- | --- | --- | --- | --- |
| a) Watching TV or a video |  |  |  |  |  |
| b) Playing indoor games |  |  |  |  |  |
| c) Eating a meal |  |  |  |  |  |
| d) Going for a walk |  |  |  |  |  |
| e) Going places |  |  |  |  |  |
| f) Visiting friends or relatives |  |  |  |  |  |
| g) Playing sports |  |  |  |  |  |
| h) Sitting and talking about things |  |  |  |  |  |

**Fathers’ questionnaire**

Do you smoke tobacco at present?

1. Yes
2. No

If you smoke, please indicate, how often?

1. Every day
2. About 2-3 times a week
3. About once a week
4. About once a month
5. About once a year

How often do you participate in parties, where you drink alcohol, even if just the least amount of it?

1. Yes, almost every day
2. About 2-3 times a week
3. About once a week
4. About once a month
5. About once a year
6. Never.

**Mothers’ questionnaire**

Do you smoke tobacco at present?

1. Yes
2. No

If you smoke, please indicate, how often?

1. Every day
2. About 2-3 times a week
3. About once a week
4. About once a month
5. About once a year

How often do you participate in parties, where you drink alcohol, even if just the least amount of it?

1. Yes, almost every day
2. About 2-3 times a week
3. About once a week
4. About once a month
5. About once a year
6. Never.
